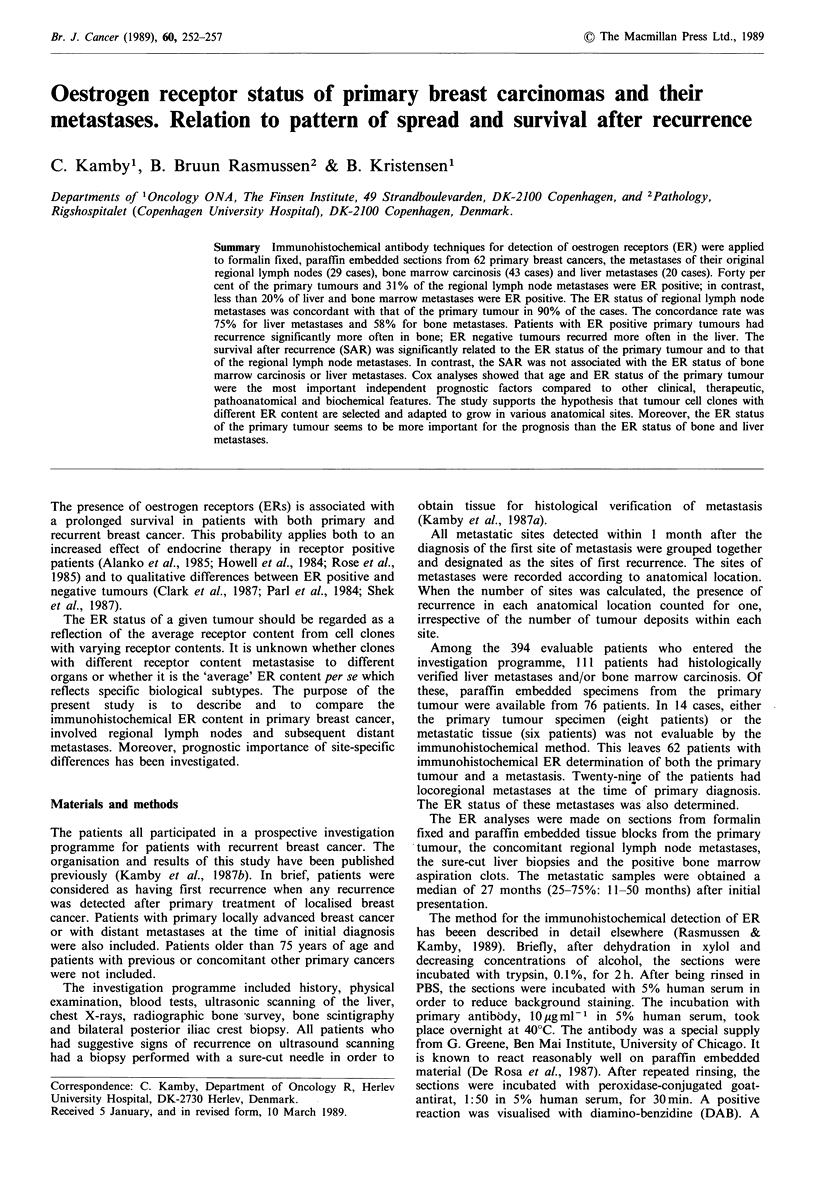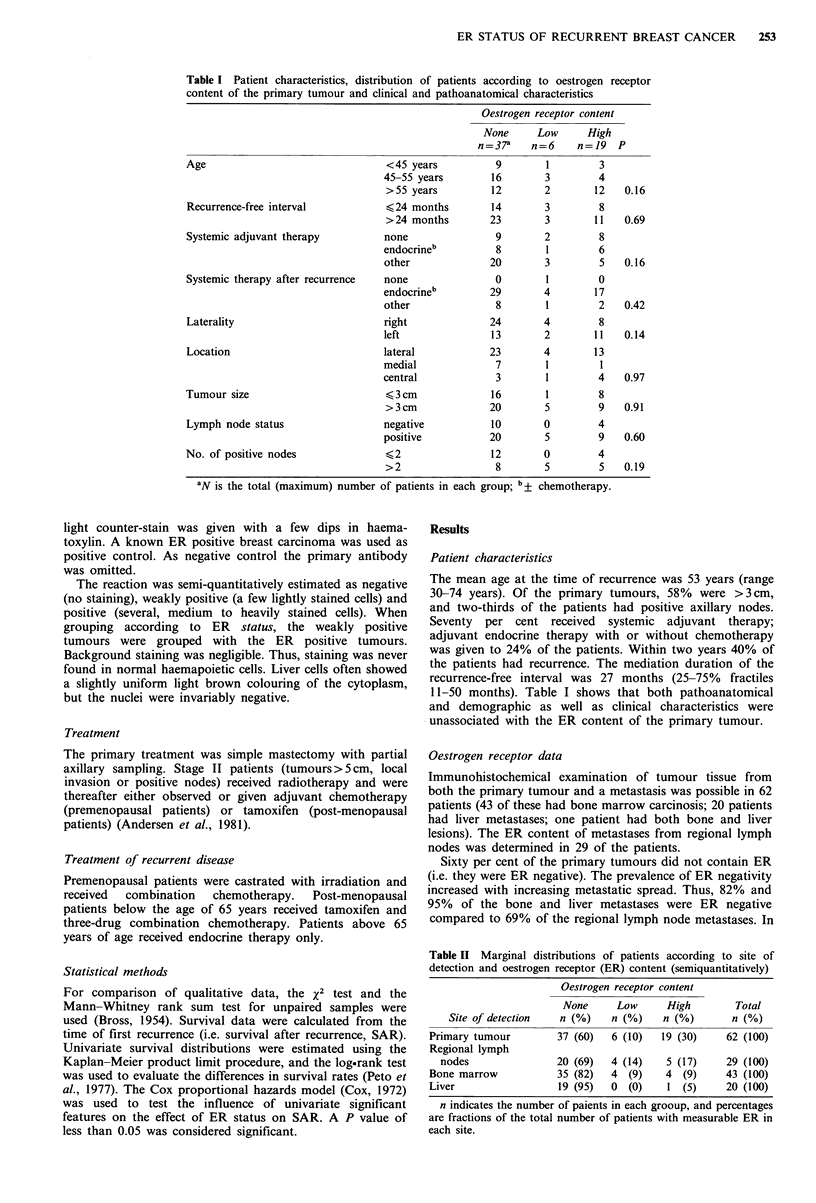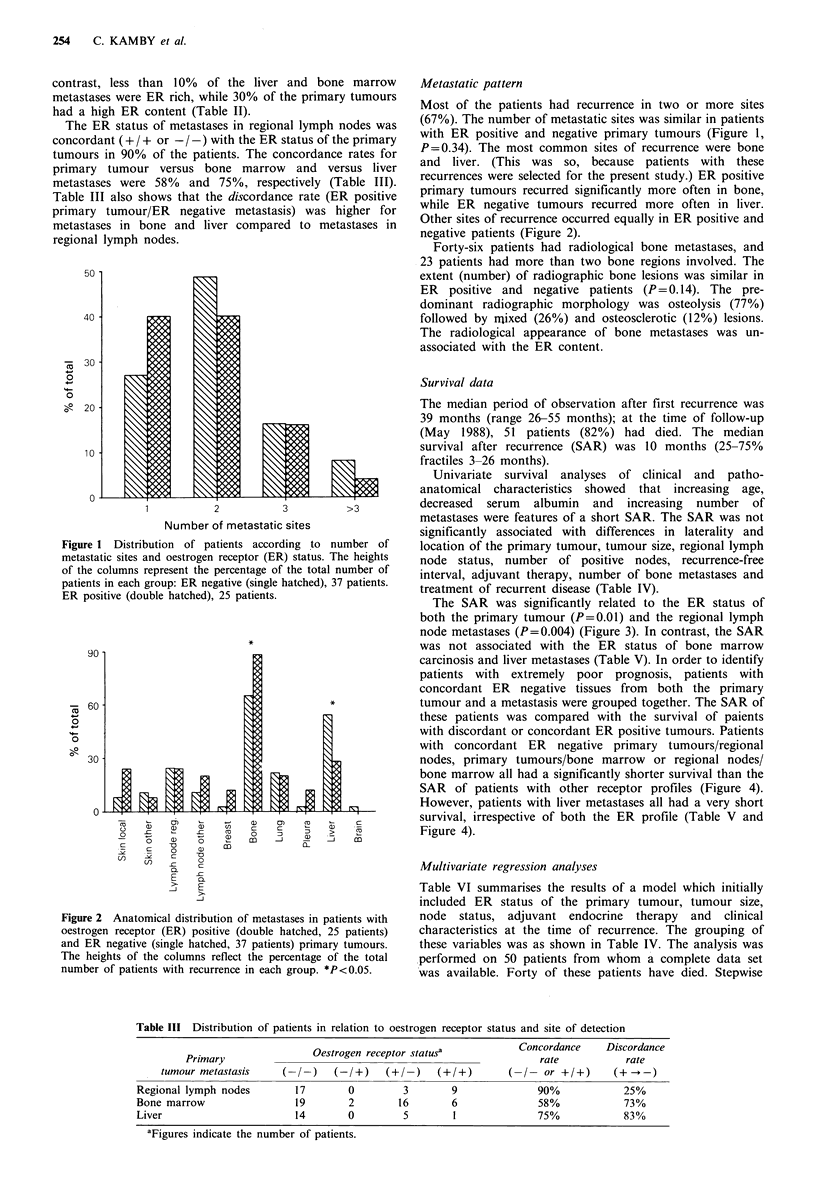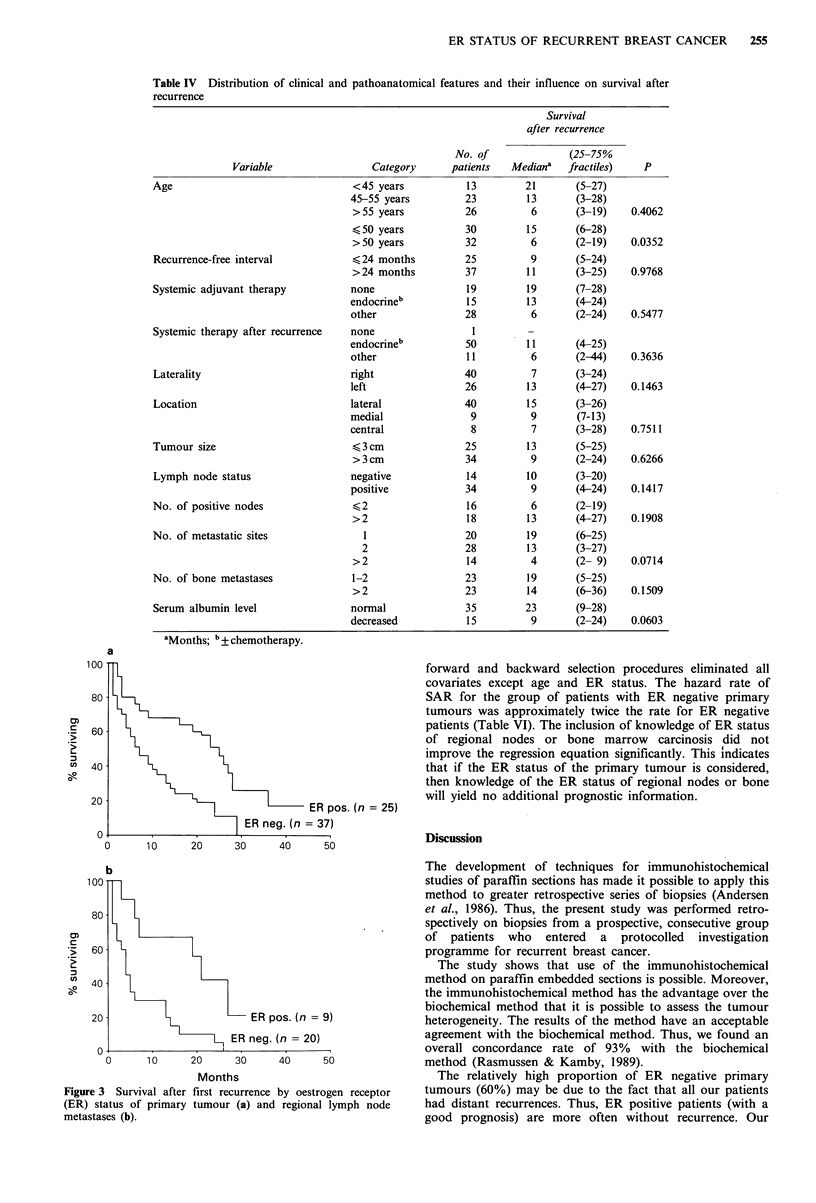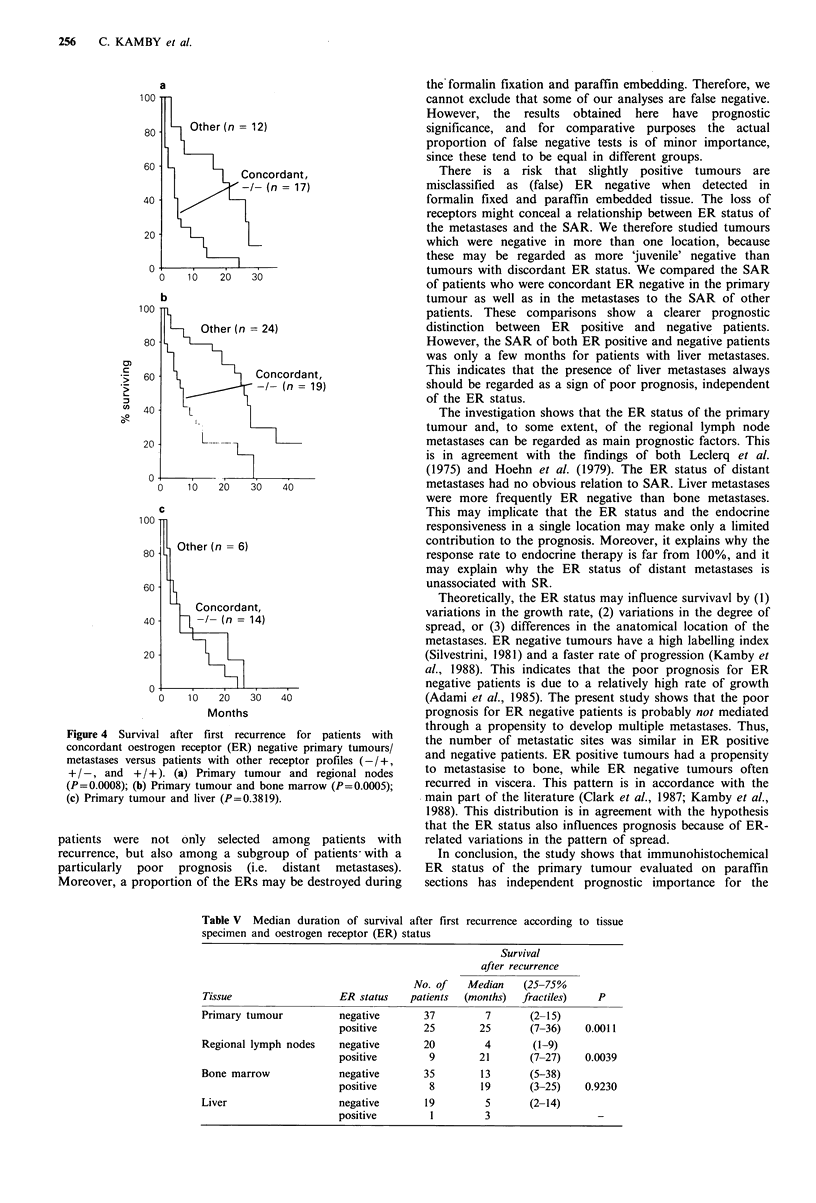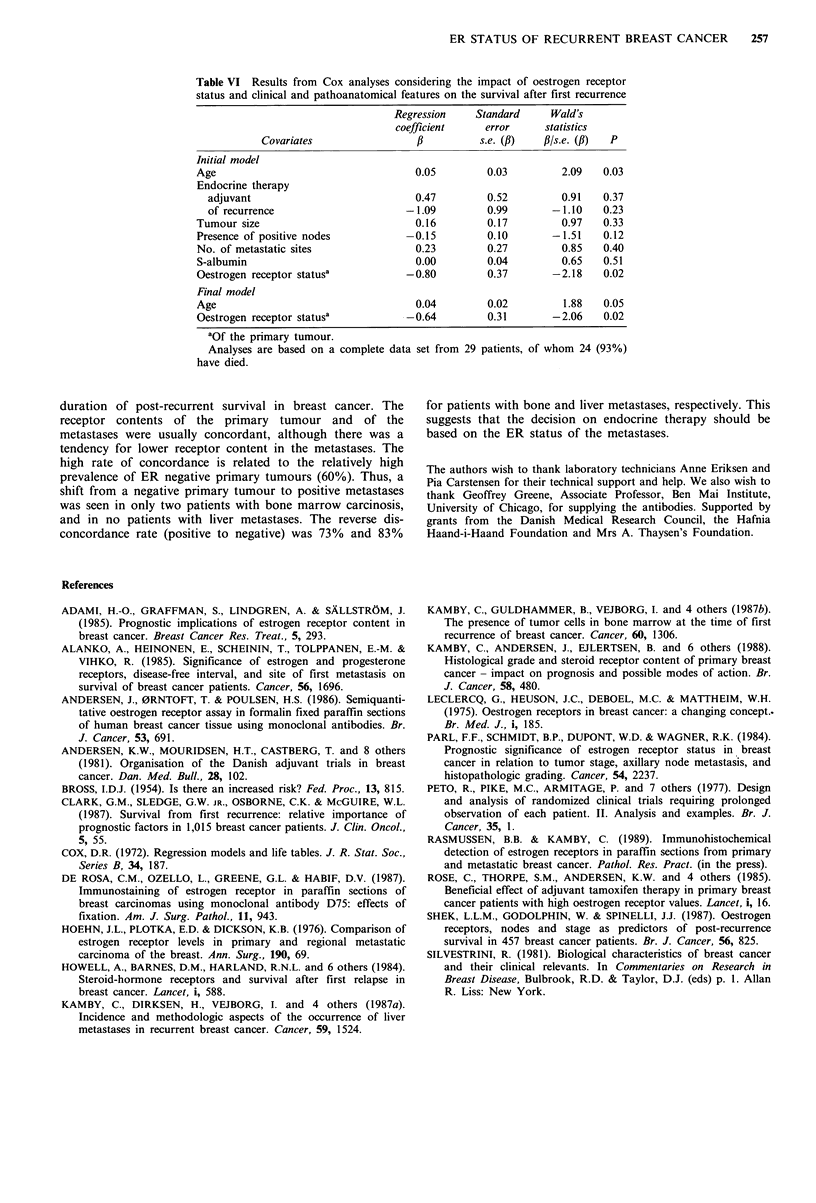# Oestrogen receptor status of primary breast carcinomas and their metastases. Relation to pattern of spread and survival after recurrence.

**DOI:** 10.1038/bjc.1989.264

**Published:** 1989-08

**Authors:** C. Kamby, B. B. Rasmussen, B. Kristensen

**Affiliations:** Department of Oncology ONA, Finsen Institute, Copenhagen, Denmark.

## Abstract

Immunohistochemical antibody techniques for detection of oestrogen receptors (ER) were applied to formalin fixed, paraffin embedded sections from 62 primary breast cancers, the metastases of their original regional lymph nodes (29 cases), bone marrow carcinosis (43 cases) and liver metastases (20 cases). Forty per cent of the primary tumours and 31% of the regional lymph node metastases were ER positive; in contrast, less than 20% of liver and bone marrow metastases were ER positive. The ER status of regional lymph node metastases was concordant with that of the primary tumour in 90% of the cases. The concordance rate was 75% for liver metastases and 58% for bone metastases. Patients with ER positive primary tumours had recurrence significantly more often in bone; ER negative tumours recurred more often in the liver. The survival after recurrence (SAR) was significantly related to the ER status of the primary tumour and to that of the regional lymph node metastases. In contrast, the SAR was not associated with the ER status of bone marrow carcinosis or liver metastases. Cox analyses showed that age and ER status of the primary tumour were the most important independent prognostic factors compared to other clinical, therapeutic, pathoanatomical and biochemical features. The study supports the hypothesis that tumour cell clones with different ER content are selected and adapted to grow in various anatomical sites. Moreover, the ER status of the primary tumour seems to be more important for the prognosis than the ER status of bone and liver metastases.


Br. J. Cancer (1989), 60, 252–257 © The Macmillan Press Ltd., 1989

### Oestrogen receptor status of primary breast carcinomas and their metastases. Relation to pattern of spread and survival after recurrence

C. Kamby[1], B. Bruun Rasmussen[2] & B. Kristensen[1]

*Departments of [1]Oncology ONA, The Finsen Institute, 49 Strandboulevarden, DK-2100 Copenhagen, and [2]Pathology, Rigshospitalet (Copenhagen University Hospital), DK-2100 Copenhagen, Denmark.*

**Summary** Immunohistochemical antibody techniques for detection of oestrogen receptors (ER) were applied to formalin fixed, paraffin embedded sections from 62 primary breast cancers, the metastases of their original regional lymph nodes (29 cases), bone marrow carcinosis (43 cases) and liver metastases (20 cases). Forty per cent of the primary tumours and 31% of the regional lymph node metastases were ER positive; in contrast, less than 20% of liver and bone marrow metastases were ER positive. The ER status of regional lymph node metastases was concordant with that of the primary tumour in 90% of the cases. The concordance rate was 75% for liver metastases and 58% for bone metastases. Patients with ER positive primary tumours had recurrence significantly more often in bone; ER negative tumours recurred more often in the liver. The survival after recurrence (SAR) was significantly related to the ER status of the primary tumour and to that of the regional lymph node metastases. In contrast, the SAR was not associated with the ER status of bone marrow carcinosis or liver metastases. Cox analyses showed that age and ER status of the primary tumour were the most important independent prognostic factors compared to other clinical, therapeutic, pathoanatomical and biochemical features. The study supports the hypothesis that tumour cell clones with different ER content are selected and adapted to grow in various anatomical sites. Moreover, the ER status of the primary tumour seems to be more important for the prognosis than the ER status of bone and liver metastases.

The presence of oestrogen receptors (ERs) is associated with a prolonged survival in patients with both primary and recurrent breast cancer. This probability applies both to an increased effect of endocrine therapy in receptor positive patients (Alanko *et al.*, 1985; Howell *et al.*, 1984; Rose *et al.*, 1985) and to qualitative differences between ER positive and negative tumours (Clark *et al.*, 1987; Parl *et al.*, 1984; Shek *et al.*, 1987).

The ER status of a given tumour should be regarded as a reflection of the average receptor content from cell clones with varying receptor contents. It is unknown whether clones with different receptor content metastasise to different organs or whether it is the 'average' ER content *per se* which reflects specific biological subtypes. The purpose of the present study is to describe and to compare the immunohistochemical ER content in primary breast cancer, involved regional lymph nodes and subsequent distant metastases. Moreover, prognostic importance of site-specific differences has been investigated.

### Materials and methods

The patients all participated in a prospective investigation programme for patients with recurrent breast cancer. The organisation and results of this study have been published previously (Kamby *et al.*, 1987*b*). In brief, patients were considered as having first recurrence when any recurrence was detected after primary treatment of localised breast cancer. Patients with primary locally advanced breast cancer or with distant metastases at the time of initial diagnosis were also included. Patients older than 75 years of age and patients with previous or concomitant other primary cancers were not included.

The investigation programme included history, physical examination, blood tests, ultrasonic scanning of the liver, chest X-rays, radiographic bone survey, bone scintigraphy and bilateral posterior iliac crest biopsy. All patients who had suggestive signs of recurrence on ultrasound scanning had a biopsy performed with a sure-cut needle in order to obtain tissue for histological verification of metastasis (Kamby *et al.*, 1987*a*).

All metastatic sites detected within 1 month after the diagnosis of the first site of metastasis were grouped together and designated as the sites of first recurrence. The sites of metastases were recorded according to anatomical location. When the number of sites was calculated, the presence of recurrence in each anatomical location counted for one, irrespective of the number of tumour deposits within each site.

Among the 394 evaluable patients who entered the investigation programme, 111 patients had histologically verified liver metastases and/or bone marrow carcinosis. Of these, paraffin embedded specimens from the primary tumour were available from 76 patients. In 14 cases, either the primary tumour specimen (eight patients) or the metastatic tissue (six patients) was not evaluable by the immunohistochemical method. This leaves 62 patients with immunohistochemical ER determination of both the primary tumour and a metastasis. Twenty-nine of the patients had locoregional metastases at the time of primary diagnosis. The ER status of these metastases was also determined.

The ER analyses were made on sections from formalin fixed and paraffin embedded tissue blocks from the primary tumour, the concomitant regional lymph node metastases, the sure-cut liver biopsies and the positive bone marrow aspiration clots. The metastatic samples were obtained a median of 27 months (25–75%: 11–50 months) after initial presentation.

The method for the immunohistochemical detection of ER has beeen described in detail elsewhere (Rasmussen & Kamby, 1989). Briefly, after dehydration in xylol and decreasing concentrations of alcohol, the sections were incubated with trypsin, 0.1%, for 2 h. After being rinsed in PBS, the sections were incubated with 5% human serum in order to reduce background staining. The incubation with primary antibody, $10\,\mu g\,ml^{-1}$ in 5% human serum, took place overnight at 40°C. The antibody was a special supply from G. Greene, Ben Mai Institute, University of Chicago. It is known to react reasonably well on paraffin embedded material (De Rosa *et al.*, 1987). After repeated rinsing, the sections were incubated with peroxidase-conjugated goat-antirat, 1:50 in 5% human serum, for 30 min. A positive reaction was visualised with diamino-benzidine (DAB). A

Correspondence: C. Kamby, Department of Oncology R, Herlev University Hospital, DK-2730 Herlev, Denmark.
Received 5 January, and in revised form, 10 March 1989.

**Table I** Patient characteristics, distribution of patients according to oestrogen receptor content of the primary tumour and clinical and pathoanatomical characteristics

| | | *Oestrogen receptor content* | | | |
|---|---|---|---|---|---|
| | | *None* $n=37^a$ | *Low* $n=6$ | *High* $n=19$ | *P* |
| Age | <45 years | 9 | 1 | 3 | |
| | 45–55 years | 16 | 3 | 4 | |
| | >55 years | 12 | 2 | 12 | 0.16 |
| Recurrence-free interval | ⩽24 months | 14 | 3 | 8 | |
| | >24 months | 23 | 3 | 11 | 0.69 |
| Systemic adjuvant therapy | none | 9 | 2 | 8 | |
| | endocrine[b] | 8 | 1 | 6 | |
| | other | 20 | 3 | 5 | 0.16 |
| Systemic therapy after recurrence | none | 0 | 1 | 0 | |
| | endocrine[b] | 29 | 4 | 17 | |
| | other | 8 | 1 | 2 | 0.42 |
| Laterality | right | 24 | 4 | 8 | |
| | left | 13 | 2 | 11 | 0.14 |
| Location | lateral | 23 | 4 | 13 | |
| | medial | 7 | 1 | 1 | |
| | central | 3 | 1 | 4 | 0.97 |
| Tumour size | ⩽3 cm | 16 | 1 | 8 | |
| | >3 cm | 20 | 5 | 9 | 0.91 |
| Lymph node status | negative | 10 | 0 | 4 | |
| | positive | 20 | 5 | 9 | 0.60 |
| No. of positive nodes | ⩽2 | 12 | 0 | 4 | |
| | >2 | 8 | 5 | 5 | 0.19 |

[a]*N* is the total (maximum) number of patients in each group; [b]± chemotherapy.

light counter-stain was given with a few dips in haematoxylin. A known ER positive breast carcinoma was used as positive control. As negative control the primary antibody was omitted.

The reaction was semi-quantitatively estimated as negative (no staining), weakly positive (a few lightly stained cells) and positive (several, medium to heavily stained cells). When grouping according to ER *status*, the weakly positive tumours were grouped with the ER positive tumours. Background staining was negligible. Thus, staining was never found in normal haemapoietic cells. Liver cells often showed a slightly uniform light brown colouring of the cytoplasm, but the nuclei were invariably negative.

### *Treatment*

The primary treatment was simple mastectomy with partial axillary sampling. Stage II patients (tumours>5 cm, local invasion or positive nodes) received radiotherapy and were thereafter either observed or given adjuvant chemotherapy (premenopausal patients) or tamoxifen (post-menopausal patients) (Andersen *et al*., 1981).

### *Treatment of recurrent disease*

Premenopausal patients were castrated with irradiation and received combination chemotherapy. Post-menopausal patients below the age of 65 years received tamoxifen and three-drug combination chemotherapy. Patients above 65 years of age received endocrine therapy only.

### *Statistical methods*

For comparison of qualitative data, the $\chi^2$ test and the Mann–Whitney rank sum test for unpaired samples were used (Bross, 1954). Survival data were calculated from the time of first recurrence (i.e. survival after recurrence, SAR). Univariate survival distributions were estimated using the Kaplan–Meier product limit procedure, and the log•rank test was used to evaluate the differences in survival rates (Peto *et al*., 1977). The Cox proportional hazards model (Cox, 1972) was used to test the influence of univariate significant features on the effect of ER status on SAR. A *P* value of less than 0.05 was considered significant.

### Results

### *Patient characteristics*

The mean age at the time of recurrence was 53 years (range 30–74 years). Of the primary tumours, 58% were >3 cm, and two-thirds of the patients had positive axillary nodes. Seventy per cent received systemic adjuvant therapy; adjuvant endocrine therapy with or without chemotherapy was given to 24% of the patients. Within two years 40% of the patients had recurrence. The mediation duration of the recurrence-free interval was 27 months (25–75% fractiles 11–50 months). Table I shows that both pathoanatomical and demographic as well as clinical characteristics were unassociated with the ER content of the primary tumour.

### *Oestrogen receptor data*

Immunohistochemical examination of tumour tissue from both the primary tumour and a metastasis was possible in 62 patients (43 of these had bone marrow carcinosis; 20 patients had liver metastases; one patient had both bone and liver lesions). The ER content of metastases from regional lymph nodes was determined in 29 of the patients.

Sixty per cent of the primary tumours did not contain ER (i.e. they were ER negative). The prevalence of ER negativity increased with increasing metastatic spread. Thus, 82% and 95% of the bone and liver metastases were ER negative compared to 69% of the regional lymph node metastases. In

**Table II** Marginal distributions of patients according to site of detection and oestrogen receptor (ER) content (semiquantitatively)

| | *Oestrogen receptor content* | | | |
|---|---|---|---|---|
| *Site of detection* | *None* *n* (%) | *Low* *n* (%) | *High* *n* (%) | *Total* *n* (%) |
| Primary tumour | 37 (60) | 6 (10) | 19 (30) | 62 (100) |
| Regional lymph nodes | 20 (69) | 4 (14) | 5 (17) | 29 (100) |
| Bone marrow | 35 (82) | 4 (9) | 4 (9) | 43 (100) |
| Liver | 19 (95) | 0 (0) | 1 (5) | 20 (100) |

*n* indicates the number of paients in each grooup, and percentages are fractions of the total number of patients with measurable ER in each site.

contrast, less than 10% of the liver and bone marrow metastases were ER rich, while 30% of the primary tumours had a high ER content (Table II).

The ER status of metastases in regional lymph nodes was concordant (+/+ or −/−) with the ER status of the primary tumours in 90% of the patients. The concordance rates for primary tumour versus bone marrow and versus liver metastases were 58% and 75%, respectively (Table III). Table III also shows that the *dis*cordance rate (ER positive primary tumour/ER negative metastasis) was higher for metastases in bone and liver compared to metastases in regional lymph nodes.

**Figure 1** Distribution of patients according to number of metastatic sites and oestrogen receptor (ER) status. The heights of the columns represent the percentage of the total number of patients in each group: ER negative (single hatched), 37 patients. ER positive (double hatched), 25 patients.

**Figure 2** Anatomical distribution of metastases in patients with oestrogen receptor (ER) positive (double hatched, 25 patients) and ER negative (single hatched, 37 patients) primary tumours. The heights of the columns reflect the percentage of the total number of patients with recurrence in each group. $*P<0.05$.

*Metastatic pattern*

Most of the patients had recurrence in two or more sites (67%). The number of metastatic sites was similar in patients with ER positive and negative primary tumours (Figure 1, $P=0.34$). The most common sites of recurrence were bone and liver. (This was so, because patients with these recurrences were selected for the present study.) ER positive primary tumours recurred significantly more often in bone, while ER negative tumours recurred more often in liver. Other sites of recurrence occurred equally in ER positive and negative patients (Figure 2).

Forty-six patients had radiological bone metastases, and 23 patients had more than two bone regions involved. The extent (number) of radiographic bone lesions was similar in ER positive and negative patients ($P=0.14$). The predominant radiographic morphology was osteolysis (77%) followed by mixed (26%) and osteosclerotic (12%) lesions. The radiological appearance of bone metastases was unassociated with the ER content.

*Survival data*

The median period of observation after first recurrence was 39 months (range 26–55 months); at the time of follow-up (May 1988), 51 patients (82%) had died. The median survival after recurrence (SAR) was 10 months (25–75% fractiles 3–26 months).

Univariate survival analyses of clinical and pathoanatomical characteristics showed that increasing age, decreased serum albumin and increasing number of metastases were features of a short SAR. The SAR was not significantly associated with differences in laterality and location of the primary tumour, tumour size, regional lymph node status, number of positive nodes, recurrence-free interval, adjuvant therapy, number of bone metastases and treatment of recurrent disease (Table IV).

The SAR was significantly related to the ER status of both the primary tumour ($P=0.01$) and the regional lymph node metastases ($P=0.004$) (Figure 3). In contrast, the SAR was not associated with the ER status of bone marrow carcinosis and liver metastases (Table V). In order to identify patients with extremely poor prognosis, patients with concordant ER negative tissues from both the primary tumour and a metastasis were grouped together. The SAR of these patients was compared with the survival of paients with discordant or concordant ER positive tumours. Patients with concordant ER negative primary tumours/regional nodes, primary tumours/bone marrow or regional nodes/bone marrow all had a significantly shorter survival than the SAR of patients with other receptor profiles (Figure 4). However, patients with liver metastases all had a very short survival, irrespective of both the ER profile (Table V and Figure 4).

*Multivariate regression analyses*

Table VI summarises the results of a model which initially included ER status of the primary tumour, tumour size, node status, adjuvant endocrine therapy and clinical characteristics at the time of recurrence. The grouping of these variables was as shown in Table IV. The analysis was performed on 50 patients from whom a complete data set was available. Forty of these patients have died. Stepwise

**Table III** Distribution of patients in relation to oestrogen receptor status and site of detection

| Primary tumour metastasis | Oestrogen receptor status[a] (−/−) | (−/+) | (+/−) | (+/+) | Concordance rate (−/− or +/+) | Discordance rate (+ → −) |
|---|---|---|---|---|---|---|
| Regional lymph nodes | 17 | 0 | 3 | 9 | 90% | 25% |
| Bone marrow | 19 | 2 | 16 | 6 | 58% | 73% |
| Liver | 14 | 0 | 5 | 1 | 75% | 83% |

[a]Figures indicate the number of patients.

**Table IV** Distribution of clinical and pathoanatomical features and their influence on survival after recurrence

| *Variable* | *Category* | *No. of patients* | *Survival after recurrence* *Median*[a] | *(25–75% fractiles)* | *P* |
|---|---|---|---|---|---|
| Age | <45 years | 13 | 21 | (5–27) | |
| | 45–55 years | 23 | 13 | (3–28) | |
| | >55 years | 26 | 6 | (3–19) | 0.4062 |
| | ⩽50 years | 30 | 15 | (6–28) | |
| | >50 years | 32 | 6 | (2–19) | 0.0352 |
| Recurrence-free interval | ⩽24 months | 25 | 9 | (5–24) | |
| | >24 months | 37 | 11 | (3–25) | 0.9768 |
| Systemic adjuvant therapy | none | 19 | 19 | (7–28) | |
| | endocrine[b] | 15 | 13 | (4–24) | |
| | other | 28 | 6 | (2–24) | 0.5477 |
| Systemic therapy after recurrence | none | 1 | – | | |
| | endocrine[b] | 50 | 11 | (4–25) | |
| | other | 11 | 6 | (2–44) | 0.3636 |
| Laterality | right | 40 | 7 | (3–24) | |
| | left | 26 | 13 | (4–27) | 0.1463 |
| Location | lateral | 40 | 15 | (3–26) | |
| | medial | 9 | 9 | (7-13) | |
| | central | 8 | 7 | (3–28) | 0.7511 |
| Tumour size | ⩽3 cm | 25 | 13 | (5–25) | |
| | >3 cm | 34 | 9 | (2–24) | 0.6266 |
| Lymph node status | negative | 14 | 10 | (3–20) | |
| | positive | 34 | 9 | (4–24) | 0.1417 |
| No. of positive nodes | ⩽2 | 16 | 6 | (2–19) | |
| | >2 | 18 | 13 | (4–27) | 0.1908 |
| No. of metastatic sites | 1 | 20 | 19 | (6–25) | |
| | 2 | 28 | 13 | (3–27) | |
| | >2 | 14 | 4 | (2– 9) | 0.0714 |
| No. of bone metastases | 1-2 | 23 | 19 | (5–25) | |
| | >2 | 23 | 14 | (6–36) | 0.1509 |
| Serum albumin level | normal | 35 | 23 | (9–28) | |
| | decreased | 15 | 9 | (2–24) | 0.0603 |

[a]Months; [b]±chemotherapy.

**Figure 3** Survival after first recurrence by oestrogen receptor (ER) status of primary tumour (**a**) and regional lymph node metastases (**b**).

forward and backward selection procedures eliminated all covariates except age and ER status. The hazard rate of SAR for the group of patients with ER negative primary tumours was approximately twice the rate for ER negative patients (Table VI). The inclusion of knowledge of ER status of regional nodes or bone marrow carcinosis did not improve the regression equation significantly. This indicates that if the ER status of the primary tumour is considered, then knowledge of the ER status of regional nodes or bone will yield no additional prognostic information.

### Discussion

The development of techniques for immunohistochemical studies of paraffin sections has made it possible to apply this method to greater retrospective series of biopsies (Andersen *et al.*, 1986). Thus, the present study was performed retrospectively on biopsies from a prospective, consecutive group of patients who entered a protocolled investigation programme for recurrent breast cancer.

The study shows that use of the immunohistochemical method on paraffin embedded sections is possible. Moreover, the immunohistochemical method has the advantage over the biochemical method that it is possible to assess the tumour heterogeneity. The results of the method have an acceptable agreement with the biochemical method. Thus, we found an overall concordance rate of 93% with the biochemical method (Rasmussen & Kamby, 1989).

The relatively high proportion of ER negative primary tumours (60%) may be due to the fact that all our patients had distant recurrences. Thus, ER positive patients (with a good prognosis) are more often without recurrence. Our

**Figure 4** Survival after first recurrence for patients with concordant oestrogen receptor (ER) negative primary tumours/metastases versus patients with other receptor profiles (−/+, +/−, and +/+). (**a**) Primary tumour and regional nodes ($P$=0.0008); (**b**) Primary tumour and bone marrow ($P$=0.0005); (**c**) Primary tumour and liver ($P$=0.3819).

patients were not only selected among patients with recurrence, but also among a subgroup of patients with a particularly poor prognosis (i.e. distant metastases). Moreover, a proportion of the ERs may be destroyed during the formalin fixation and paraffin embedding. Therefore, we cannot exclude that some of our analyses are false negative. However, the results obtained here have prognostic significance, and for comparative purposes the actual proportion of false negative tests is of minor importance, since these tend to be equal in different groups.

There is a risk that slightly positive tumours are misclassified as (false) ER negative when detected in formalin fixed and paraffin embedded tissue. The loss of receptors might conceal a relationship between ER status of the metastases and the SAR. We therefore studied tumours which were negative in more than one location, because these may be regarded as more 'juvenile' negative than tumours with discordant ER status. We compared the SAR of patients who were concordant ER negative in the primary tumour as well as in the metastases to the SAR of other patients. These comparisons show a clearer prognostic distinction between ER positive and negative patients. However, the SAR of both ER positive and negative patients was only a few months for patients with liver metastases. This indicates that the presence of liver metastases always should be regarded as a sign of poor prognosis, independent of the ER status.

The investigation shows that the ER status of the primary tumour and, to some extent, of the regional lymph node metastases can be regarded as main prognostic factors. This is in agreement with the findings of both Leclerq *et al.* (1975) and Hoehn *et al.* (1979). The ER status of distant metastases had no obvious relation to SAR. Liver metastases were more frequently ER negative than bone metastases. This may implicate that the ER status and the endocrine responsiveness in a single location may make only a limited contribution to the prognosis. Moreover, it explains why the response rate to endocrine therapy is far from 100%, and it may explain why the ER status of distant metastases is unassociated with SR.

Theoretically, the ER status may influence survivavl by (1) variations in the growth rate, (2) variations in the degree of spread, or (3) differences in the anatomical location of the metastases. ER negative tumours have a high labelling index (Silvestrini, 1981) and a faster rate of progression (Kamby *et al.*, 1988). This indicates that the poor prognosis for ER negative patients is due to a relatively high rate of growth (Adami *et al.*, 1985). The present study shows that the poor prognosis for ER negative patients is probably *not* mediated through a propensity to develop multiple metastases. Thus, the number of metastatic sites was similar in ER positive and negative patients. ER positive tumours had a propensity to metastasise to bone, while ER negative tumours often recurred in viscera. This pattern is in accordance with the main part of the literature (Clark *et al.*, 1987; Kamby *et al.*, 1988). This distribution is in agreement with the hypothesis that the ER status also influences prognosis because of ER-related variations in the pattern of spread.

In conclusion, the study shows that immunohistochemical ER status of the primary tumour evaluated on paraffin sections has independent prognostic importance for the

**Table V** Median duration of survival after first recurrence according to tissue specimen and oestrogen receptor (ER) status

| *Tissue* | *ER status* | *No. of patients* | *Survival after recurrence* *Median (months)* | *(25–75% fractiles)* | *P* |
|---|---|---|---|---|---|
| Primary tumour | negative | 37 | 7 | (2–15) | |
| | positive | 25 | 25 | (7–36) | 0.0011 |
| Regional lymph nodes | negative | 20 | 4 | (1–9) | |
| | positive | 9 | 21 | (7–27) | 0.0039 |
| Bone marrow | negative | 35 | 13 | (5–38) | |
| | positive | 8 | 19 | (3–25) | 0.9230 |
| Liver | negative | 19 | 5 | (2–14) | |
| | positive | 1 | 3 | | – |

**Table VI** Results from Cox analyses considering the impact of oestrogen receptor status and clinical and pathoanatomical features on the survival after first recurrence

| *Covariates* | *Regression coefficient* $\beta$ | *Standard error* *s.e.* ($\beta$) | *Wald's statistics* $\beta$/*s.e.* ($\beta$) | *P* |
|---|---|---|---|---|
| *Initial model* | | | | |
| Age | 0.05 | 0.03 | 2.09 | 0.03 |
| Endocrine therapy | | | | |
| adjuvant | 0.47 | 0.52 | 0.91 | 0.37 |
| of recurrence | −1.09 | 0.99 | −1.10 | 0.23 |
| Tumour size | 0.16 | 0.17 | 0.97 | 0.33 |
| Presence of positive nodes | −0.15 | 0.10 | −1.51 | 0.12 |
| No. of metastatic sites | 0.23 | 0.27 | 0.85 | 0.40 |
| S-albumin | 0.00 | 0.04 | 0.65 | 0.51 |
| Oestrogen receptor status[a] | −0.80 | 0.37 | −2.18 | 0.02 |
| *Final model* | | | | |
| Age | 0.04 | 0.02 | 1.88 | 0.05 |
| Oestrogen receptor status[a] | −0.64 | 0.31 | −2.06 | 0.02 |

[a]Of the primary tumour.

Analyses are based on a complete data set from 29 patients, of whom 24 (93%) have died.

duration of post-recurrent survival in breast cancer. The receptor contents of the primary tumour and of the metastases were usually concordant, although there was a tendency for lower receptor content in the metastases. The high rate of concordance is related to the relatively high prevalence of ER negative primary tumours (60%). Thus, a shift from a negative primary tumour to positive metastases was seen in only two patients with bone marrow carcinosis, and in no patients with liver metastases. The reverse disconcordance rate (positive to negative) was 73% and 83% for patients with bone and liver metastases, respectively. This suggests that the decision on endocrine therapy should be based on the ER status of the metastases.

The authors wish to thank laboratory technicians Anne Eriksen and Pia Carstensen for their technical support and help. We also wish to thank Geoffrey Greene, Associate Professor, Ben Mai Institute, University of Chicago, for supplying the antibodies. Supported by grants from the Danish Medical Research Council, the Hafnia Haand-i-Haand Foundation and Mrs A. Thaysen's Foundation.

### References

ADAMI, H.-O., GRAFFMAN, S., LINDGREN, A. & SÄLLSTRÖM, J. (1985). Prognostic implications of estrogen receptor content in breast cancer. *Breast Cancer Res. Treat.*, **5**, 293.

ALANKO, A., HEINONEN, E., SCHEININ, T., TOLPPANEN, E.-M. & VIHKO, R. (1985). Significance of estrogen and progesterone receptors, disease-free interval, and site of first metastasis on survival of breast cancer patients. *Cancer*, **56**, 1696.

ANDERSEN, J., ØRNTOFT, T. & POULSEN, H.S. (1986). Semiquantitative oestrogen receptor assay in formalin fixed paraffin sections of human breast cancer tissue using monoclonal antibodies. *Br. J. Cancer*, **53**, 691.

ANDERSEN, K.W., MOURIDSEN, H.T., CASTBERG, T. and 8 others (1981). Organisation of the Danish adjuvant trials in breast cancer. *Dan. Med. Bull.*, **28**, 102.

BROSS, I.D.J. (1954). Is there an increased risk? *Fed. Proc.*, **13**, 815.

CLARK, G.M., SLEDGE, G.W. JR., OSBORNE, C.K. & McGUIRE, W.L. (1987). Survival from first recurrence: relative importance of prognostic factors in 1,015 breast cancer patients. *J. Clin. Oncol.*, **5**, 55.

COX, D.R. (1972). Regression models and life tables. *J. R. Stat. Soc., Series B*, **34**, 187.

DE ROSA, C.M., OZELLO, L., GREENE, G.L. & HABIF, D.V. (1987). Immunostaining of estrogen receptor in paraffin sections of breast carcinomas using monoclonal antibody D75: effects of fixation. *Am. J. Surg. Pathol.*, **11**, 943.

HOEHN, J.L., PLOTKA, E.D. & DICKSON, K.B. (1976). Comparison of estrogen receptor levels in primary and regional metastatic carcinoma of the breast. *Ann. Surg.*, **190**, 69.

HOWELL, A., BARNES, D.M., HARLAND, R.N.L. and 6 others (1984). Steroid-hormone receptors and survival after first relapse in breast cancer. *Lancet*, **i**, 588.

KAMBY, C., DIRKSEN, H., VEJBORG, I. and 4 others (1987*a*). Incidence and methodologic aspects of the occurrence of liver metastases in recurrent breast cancer. *Cancer*, **59**, 1524.

KAMBY, C., GULDHAMMER, B., VEJBORG, I. and 4 others (1987*b*). The presence of tumor cells in bone marrow at the time of first recurrence of breast cancer. *Cancer*, **60**, 1306.

KAMBY, C., ANDERSEN, J., EJLERTSEN, B. and 6 others (1988). Histological grade and steroid receptor content of primary breast cancer – impact on prognosis and possible modes of action. *Br. J. Cancer*, **58**, 480.

LECLERCQ, G., HEUSON, J.C., DEBOEL, M.C. & MATTHEIM, W.H. (1975). Oestrogen receptors in breast cancer: a changing concept. *Br. Med. J.*, **i**, 185.

PARL, F.F., SCHMIDT, B.P., DUPONT, W.D. & WAGNER, R.K. (1984). Prognostic significance of estrogen receptor status in breast cancer in relation to tumor stage, axillary node metastasis, and histopathologic grading. *Cancer*, **54**, 2237.

PETO, R., PIKE, M.C., ARMITAGE, P. and 7 others (1977). Design and analysis of randomized clinical trials requiring prolonged observation of each patient. II. Analysis and examples. *Br. J. Cancer*, **35**, 1.

RASMUSSEN, B.B. & KAMBY, C. (1989). Immunohistochemical detection of estrogen receptors in paraffin sections from primary and metastatic breast cancer. *Pathol. Res. Pract.* (in the press).

ROSE, C., THORPE, S.M., ANDERSEN, K.W. and 4 others (1985). Beneficial effect of adjuvant tamoxifen therapy in primary breast cancer patients with high oestrogen receptor values. *Lancet*, **i**, 16.

SHEK, L.L.M., GODOLPHIN, W. & SPINELLI, J.J. (1987). Oestrogen receptors, nodes and stage as predictors of post-recurrence survival in 457 breast cancer patients. *Br. J. Cancer*, **56**, 825.

SILVESTRINI, R. (1981). Biological characteristics of breast cancer and their clinical relevants. In *Commentaries on Research in Breast Disease*, Bulbrook, R.D. & Taylor, D.J. (eds) p. 1. Allan R. Liss: New York.